# Pharmacogenomics landscape of COVID-19 therapy response in Serbian population and comparison with worldwide populations

**DOI:** 10.5937/jomb0-26725

**Published:** 2020-10-02

**Authors:** Biljana Stanković, Nikola Kotur, Vladimir Gašić, Kristel Klaassen, Bojan Ristivojević, Maja Stojiljković, Sonja Pavlović, Branka Zukić

**Affiliations:** 1 University of Belgrade, Institute of Molecular Genetics and Genetic Engineering, Laboratory for Molecular Biomedicine, Belgrade

**Keywords:** azithromycin, chloroquine/hydroxychloroquine, COVID-19, lopinavir, pharmacogenomics markers, population pharmacogenomics, ritonavir, ritonavir, populaciona farmakogenomika, lopinavir, hlorokin/hidroksihlorokin, farmakogenomski markeri, COVID-19, azitromicin

## Abstract

**Background:**

Since there are no certified therapeutics to treat COVID-19 patients, drug repurposing became important. With lack of time to test individual pharmacogenomics markers, population pharmacogenomics could be helpful in predicting a higher risk of developing adverse reactions and treatment failure in COVID-19 patients. Aim of our study was to identify pharmacogenes and pharmacogenomics markers associated with drugs recommended for COVID-19 treatment, chloroquine/hydroxychloroquine, azithromycin, lopinavir and ritonavir, in population of Serbia and other world populations.

**Methods:**

Genotype information of 143 individuals of Serbian origin was extracted from database previously obtained using TruSight One Gene Panel (Illumina). Genotype data of individuals from different world populations were extracted from the 1000 Genome Project. Fisher's exact test was used for comparison of allele frequencies.

**Results:**

We have identified 11 potential pharmacogenomics markers in 7 pharmacogenes relevant for COVID-19 treatment. Based on high alternative allele frequencies in population and the functional effect of the variants, *ABCB1* rs1045642 and rs2032582 could be relevant for reduced clearance of azithromycin, lopinavir and ritonavir drugs and *UGT1A7* rs17868323 for hyperbilirubinemia in ritonavir treated COVID-19 patients in Serbian population. *SLCO1B1* rs4149056 is a potential marker of lopinavir response, especially in Italian population. Our results confirmed that pharmacogenomics profile of African population is different from the rest of the world.

**Conclusions:**

Considering population specific pharmacogenomics landscape, preemptive testing for pharmacogenes relevant for drugs used in COVID-19 treatment could contribute to better understanding of the inconsistency in therapy response and could be applied to improve the outcome of the COVID-19 patients.

## Abstract (sr)

**Uvod:**

Kako ne postoje odobreni terapeutici za lečenje pacijenata sa COVID-19, mogućnost upotrebe postojećih lekova je postala važna. U nedostatku vremena za testiranje farmakogenomskih markera kod pojedinaca, populaciona farmakogenomika bi mogla biti od koristi u predviđanju povećanog rizika za pojavu neželjenih reakcija i neuspeha lečenja kod pacijenata sa COVID-19. Cilj naše studije bio je identifikovanje farmakogena i farmakogenomskih markera povezanih sa lekovima koji se preporučuju za lečenje COVID-19, hlorokin/hidroksihlorokin, azitromicin, lopinavir i ritonavir, u populaciji Srbije i drugim svetskim populacijama.

**Metode:**

Podaci o genotipu 143 osobe srpskog porekla dobijeni su iz baze podataka prethodno formirane analizama genoma korišćenjem TruSight One Gene Panel (Illumina). Podaci o genotipu pojedinaca iz različitih svetskih populacija dobijeni su iz Projekta 1000 genoma. Fišerov egzaktni test korišćen je za poređenje učestalosti alela.

**Rezultati:**

Identifikovali smo 11 potencijalnih farmakogenomskih markera u 7 farmakogena značajnih za lečenje COVID-19. Na osnovu visoke alterativne učestalosti alela u populaciji Srbije i funkcionalnog efekta varijanti, *ABCB1* rs1045642 i rs2032582 mogu biti značajne za smanjeni klirens lekova azitromicina, lopinavira i ritonavira, a varijanta *UGT1A7* rs17868323 za hiperbilirubinemiju kod bolesnika sa COVID-19 koji se leče ritonavirom. *SLCO1B1* rs4149056 je potencijalni marker odgovora na lopinavir, posebno u populaciji Italije. Naši rezultati potvrdili su da se farmakogenomski profil afričke populacije razlikuje od ostatka sveta.

**Zaključak:**

Uzimajući u obzir farmakogenomski profil specifičan za populaciju, preventivno testiranje farmakogena značajnih za lekove koji se koriste u lečenju COVID-19 moglo bi doprineti boljem razumevanju interindividualnih razlika u odgovorima na terapiju i poboljšanju ishoda lečenja pacijenata sa COVID-19.

## Introduction

In December 2019, in the province Wuhan of the People's Republic of China, a novel virus emerged as a cause of pneumonia, termed, due to its relations with MERS-CoV and SARS-CoV, as SARS-CoV-2 [1]. Since then, the spread of SARS-CoV-2 has caused a global pandemic of the disease COVID-19 [2]. Its severity has overburdened health systems around the world, raising a call to action for finding ways to prevent it and, if possible, treat it.

By studying the life cycle of SARS-CoV-2, several drugs have been recommended for treatment of COVID-19 patients [3]. Among them, two groups of drugs, chloroquine/hydroxychloroquine and lopinavirritonavir combination, are being studied in 63 and 62 clinical trials for COVID-19 treatment, respectively [4].

Therapy that has shown the most promise is the anti-malarial drug group, comprised of chloroquine and hydroxychloroquine, which prevents viral entry and inhibits endocytosis. Chloroquine interferes with the terminal glycosylation of the cellular receptor, angiotensin-converting enzyme 2, preventing virus entry. It also inhibits replication, virus transport and release. Chloroquine has an immune-modulating activity, which may synergistically enhance its antiviral effect in vivo [5] [6]. Hydroxychloroquine is a chloroquine analogue, more soluble than chloroquine, and shares the same mechanism of action as chloroquine [7].

Azithromycin is a macrolide antibiotic. Immunocompromised patients after SARS-CoV-2 infection are susceptible to bacterial superinfection, thus, azithromycin could be beneficial in COVID-19 treatment. In addition, azithromycin has been shown to be effective in preventing severe respiratory complications in patients with viral infections [8].

The other promising drug combination for COVID-19 therapy is the antiretroviral drug group, specifically the lopinavir-ritonavir antiretroviral combination that inhibits the 3-chymotrypsin-like protease [9]. In the lopinavir-ritonavir antiretroviral combination, the main agent is lopinavir, which has previously shown to have effect against MERS-CoV *in vitro* [10] and in an animal model [11]. Ritonavir is used to inhibit the cytochrome P450 3A4 isoform [12], in order to increase the plasma concentration of antiretroviral drugs like lopinavir [13].

Besides inconsistent therapeutic effect of both drug combinations, they were commonly used in the treatment of COVID-19 patients [14] [15]. However, adverse drug reactions (ADR) have been reported for both drug combinations.

Chloroquine derivates can cause irreversible toxic retinopathy, cardiac rhythm disorders [16], gastrointestinal problems, hypoglycemia, bone marrow suppression [17] and, in glucose-6 phosphate dehydrogenase (G6PD) deficient patients, severe hemolysis [18] [19].

ADRs are also common when the lopinavir-ritonavir combination is taken [20]. It has been reported that a significant rise in transaminases is common in patients receiving this therapy [21]. Among other possible ADRs of antiretroviral drugs are dyslipidemia, gastrointestinal upset, hyperbilirubinemia, nephrolithiasis, etc. [22] [23].

It is well documented that patient's individual genomic profile could be a basis for ADRs. Pharmacogenomics, with an approach to exploit individual genomic information to predict drug efficacy and/or toxicity, could be of great benefit in adjusting COVID-19 therapy. Moreover, population-based genome analysis and implementation of principles of pharmacogenomics, considering genome variations of a population, also have the potential to maximize therapeutic benefit and to avoid ADRs.

Based on the knowledge on absorption, distribution, metabolism and excretion (ADME) of the drugs of interest, it is possible to indicate potential pharmacogenes relevant for therapy response in COVID-19 patients.

In plasma, chloroquine binds to the serum albumin and α1-acid glycoprotein. It is metabolized by CYP2C8, CYP3A4, CYP3A5, CYP2D6 and CYP1A1 enzymes. CYP2C8 can create a metabolite of chloroquine that is more efficient than the drug itself [24]. It is also well established that chloroquine-induced haemolysis is a result of reduced G6PD enzyme activity [18] [19]. Chloroquine leaves the cell via the ABCB1 transporter. All of the proteins mentioned could have an impact to chloroquine metabolism if affected with genetic variations in corresponding genes.

Azithromycin targets protein-arginine deiminase type-4 (PADI4). It is metabolized by the CYP3A4 enzyme. This antibiotic is ejected out of the cell via the ABCB1 and ABCC2 transporters. Genetic variants in *ABCB1* gene could be a cause of different therapeutic response to azithromycin [25].

The genes *ABCB1, ABCC1* and *ABCC2* encode the membrane transporters of the ATP-binding cassette transporters. They play key roles in multi-drug resistance, since they can contribute to efflux of drugs [26]. Another membrane transporter is encoded by the *SLCO1B1* and it contributes to anion transportation [27]. These transporters are important in influencing lopinavir and ritonavir plasma levels, thus implicating possible drug toxicity events.

Among the metabolizers, *UGT1A1, UGT1A3* and *UGT1A7* encode the glucuronosyltransferases, enzymes that participate in an important step of metabolization of drugs, called glucuronidation [28]. Genes *CYP1A1, CYP2C8, CYP2D6, CYP3A4* and *CYP3A5* encode the cytochrome P450 enzymes, oxidizers of drugs, thus contributing to drug clearance [29]. Since both lopinavir and ritonavir are metabolized by glucuronosyltransferases and cytochrome P450 oxidizers, these genes are also important for antiretroviral drug plasma levels, leading to possible failure in stopping virus replication.

Apolipoproteins are essential in fat metabolism and the relevant ones are encoded by pharmacogenes *APOE* and *APOC3* [30] [31]. Variants in these genes influence triglyceride and cholesterol levels, thus possibly contributing to dyslipidemia, a common adverse effect of antiretroviral drugs [22].

Therefore, the most promising pharmacogenes, relevant for therapy response in COVID-19 patients treated with chloroquine, hydroxychloroquine, azithro mycin or lopinavir and ritonavir are *SLCO1B1, ABCB1, ABCC1, ABCC2*, (transporter encoders), *UGT1A1, UGT1A3, UGT1A7, CYP1A1, CYP2C8, CYP2D6, CYP3A4, CYP3A5*, (metabolizer encoders), *APOE* and *APOC3* (lipoprotein encoders) and *G6PD*.

Variants in each of these pharmacogenes are potential pharmacogenomics (PGx) markers based on two selection approaches: they have already been reported as PGx markers in Pharmacogenomics Knowledge base (PharmGKB) [32], or it has been predicted that they impact the structure and/or function of encoded proteins.

The goal of this study was to perform a population PGx study in the population of Serbia, the first of its kind, by determining the frequency of PGx variants in the selected pharmacogenes, in order to recommend a possible adjustment of the guidelines for treating COVID-19 patients. Additionally, we aimed to perform the same analysis in European populations (Italian, Spanish, Finnish, British and Central European from the US) and in populations of East and South Asia, South and Central America and Africa.

## Material and Methods

### Subjects

One hundred and forty-three individuals (84 male) of Serbian origin were enrolled in this study. Informed consent for genetic analysis was obtained from every subject. The study was conducted in accordance with the Declaration of Helsinki. Genotype data of healthy individuals from other populations (total of 2504 subjects) were extracted from the 1000 Genomes Project (1000GP) [33].

### Variant selection

Variants in pharmacogenes related to pharmacokinetics, efficacy or toxicity of chloroquine/hydroxychloroquine, azithromycin, lopinavir and ritonavir were selected for analyses. The inclusion criteria were: 1. Assigned level of evidence for the variantdrug of interest pair at the PharmGKB; 2. In case protein deficiency is associated with drug response, variants associated with the protein deficiency were considered; 3. Genomic region the variant is located at is covered by the TruSight One (TSO) Gene Panel (Illumina, San Diego, CA, USA)

### In silico analysis

The effect of missense variants on protein function were assessed in silico using PolyPhen-2 and SIFT prediction algorithms implemented in Ensembl Variant Effect Predictor [34].

### Genotype data extraction

The genotype information for Serbian population was extracted from database obtained from previously analyzed coding regions of 4813 genes (clinical exomes) using TruSight One (TSO) Gene Panel (Illumina, San Diego, CA, USA) and the Variant Studio Data Analysis Software (version 3.0, Illumina, San Diego, CA, USA). If variant calling failed quality filter (quality score less than Q20, read depth less than 20 and in more than 5% of individuals), that variant was excluded from further analyses.

Genotype data of individuals from different European, Asian, African and American populations were extracted from 1000GP using Data Slicer tool implemented in Ensembl database [34]. Italians from Tuscany, Spaniards from Spain, Finns from Finland, British people from Great Britain and people of Central European descent from Utah, US were included. Also, 4 super-populations were also considered: South Asians, East Asians, Africans and Americans originating from Central and South American continents.

### Statistical analysis

For Hardy-Weinberg (HW) equilibrium Haldane exact test for autosomal bi-allelic, Graffelman-Weir exact test for X-chromosome and multi-allelic exact tests for multi-allelic variants was used. Those HW equilibrium tests were implemented in the Hardy Weinberg package (version 1.6.3) of the R software (version 4.0.0, R Foundation for Statistical Computing, Vienna, Austria) [35] [36]. To test the difference in allele frequency between the Serbian group and other populations, Fisher's exact test implemented in the stats package (version 4.0.0) of the R software was used. All statistical tests were two-tailed with the probability threshold of 0.05 for statistical significance.

## Results

### Pharmacogenomics profiling of Serbian population

Among selected pharmacogenes associated with pharmacokinetics, efficacy or toxicity of the most promising drugs currently tested against SARS-CoV-2 infection, e.g. chloroquine/hydroxychloroquine, azithromycin, lopinavir and ritonavir (*SLCO1B1, ABCB1, ABCC1, ABCC2, UGT1A1, UGT1A3, UGT1A7, CYP1A1, CYP2C8, CYP2D6, CYP3A4, CYP3A5, APOE, APOC3* and *G6PD*), in Serbian population we have detected PGx markers in 7 of them.

We have found PGx markers related to chloroquine/hydroxychloroquine in *G6PD* and *CYP2C8* genes, and PGx markers related to azithromycin in *ABCB1* gene. Potential PGx markers for lopinavir have been detected in *ABCB1, ABCC2* and *SLCO1B1* and for ritonavir in *ABCB1, APOE* and *UGT1A7* genes. In those 7 pharmacogenes, 11 variants were selected for further analyses. Genotype and allele frequencies of these 11 variants in Serbian population are presented in Table 1. For all variants, Hardy-Weinberg equilibrium was tested, using exact tests. Genotype frequencies of all variants except G6PD rs2230037 conformed to HW equilibrium. Given that the p value related to HW testing of the rs2230037 variant was near the threshold of 0.05 and the fact that 11 different variants were tested, which inflated the probability of a false positive result, we decided not to disregard rs2230037 variant in subsequent analyses.

**Table 1:** Genotype and alternative allele frequencies of selected variants in pharmacogenes associated with chloroquine/hydroxychloroquine, azithromycin, lopinavir and ritonavir response. a–P value referring to Hardy-Weinberg equilibrium testing. b–Level of evidence for variant association with drug efficacy or toxicity according to the Pharmacogenomics Knowledgebase (PharmGKB). c–genotype data were presented separately for female and male gender because rs2230037 variant is located at X-chromosome; d–Rare T allele of ABCB1 rs2032582 A>C/T variant was detected in combination with A allele in 1 subject and in combination with C allele in 2 subjects, as noted in brackets. alt–alternative; ref–reference; AAF–alternative allele frequency; C/HC–chloroquine/hydroxychloroquine; A–azithromycin; L–lopinavir; R–ritonavir

variant	alt allele	ref/ref	ref/alt	alt/alt	HWE^a^	AAF	drugs	Pharm GKB^b^	associated effect
G6PD rs2230037^c^	A	43	12	4	0.04	10.2%	C/HC	Level 3	hemolytic anemia
G6PD rs2230037	A	ref: 74	-	alt: 9					
CYP2C8 rs10509681	C	116	25	2	0.64	10.1%	C/HC	-	decreased clearance
CYP2C8 rs11572103	A	142	1	0	1	0.3%	C/HC	-	decreased clearance
CYP2C8 rs1058930	C	135	8	0	1	2.8%	C/HC	-	decreased clearance
ABCB1 rs1045642	G	34	75	34	0.62	50.0%	A/L/R	Level 3	decreased clearance
ABCB1 rs2032582^d^	C (T)	25	70 (1)	46 (2)	1	57.3% (1.0%)	A/L/R	Level 3	
ABCC2 rs8187710	A	122	21	0	1	7.3%	L	Level 3	decreased clearance
SLCO1B1 rs4149056	C	108	31	2	1	12.4%	L	Level 2B	decreased clearance
APOE rs429358	C	112	29	2	1	11.5%	R	Level 3	dislipidemia
APOE rs7412	T	126	17	0	1	5.9%	R	Level 3	dislipidemia
UGT1A7 rs17868323	G	22	62	59	0.47	62.9%	R	Level 3	hyperbiliru- binemia

We used three approaches to evaluate variants as PGx markers. First, we classified them by the level of evidence according to PharmGKB, then we determined how high alternative allele frequencies (AAF) of each of the variant were, and, finally, we assessed their functional effect using *in silico* prediction algorithms.

The level of evidence that correspond to association of each variant to drug response is extracted from PharmGKB database. Evidence level 1 corresponds to highest degree of certainty, while higher numbers correspond to lower degree of evidence fora variant-drug pair. Most of the PGx variants detected in Serbian population have level of evidence 3. Only variant *SLCO1B1* rs4149056 has higher degree ofevidence (2B).

Analysis of allele frequencies pointed out that there are several rare variants. Their AAF are less than 6%. Most of these rare variants are predicted to be deleterious/protein damaging (*CYP2C8* rs11572103, *CYP2C8* rs1058930, *APOE* rs7412). These PGx markers could be useful for individualization of therapy of their carriers.

However, only one variant with potentially deleterious effect, *SLCO1B1* rs4149056, was found to be more frequent (12.4%). This PGx marker is promising population-specific marker in Serbian population.

Nevertheless, for population pharmacogenomics, most relevant are variants with high frequency. In our study we have detected three PGx markers with AAF higher than 50% (*ABCB1* rs1045642, *ABCB1* rs2032582 and *UGT1A7* rs17868323). These PGx markers are the best candidates for preemptive testing when COVID-19 therapy is planned to be administered.

Our study draws attention to several PGx variants that could be taken into consideration during treatment of COVID-19 patients in Serbia. Two common variants in *ABCB1* gene (*ABCB1* rs1045642 and *ABCB1* rs2032582) could lead to reduced clearance of azithromycin, lopinavir and ritonavir drugs. Apart from *ABCB1* variants, minor alleles of *SLCO1B1* rs4149056 are also potentially associated with reduced clearance of lopinavir. Additionally, patients treated with ritonavir could experience hyperbilirubinemia if they are carriers of *UGT1A7* rs17868323 allele, whose prevalence in Serbian population is 62.9%.

### Comparative population pharmacogenomics analysis

Based on allele frequency of 11 selected variants in 7 pharmacogenes, we carried out population pharmacogenomics analysis focusing on variants common in at least one population.

**Figure 1:**
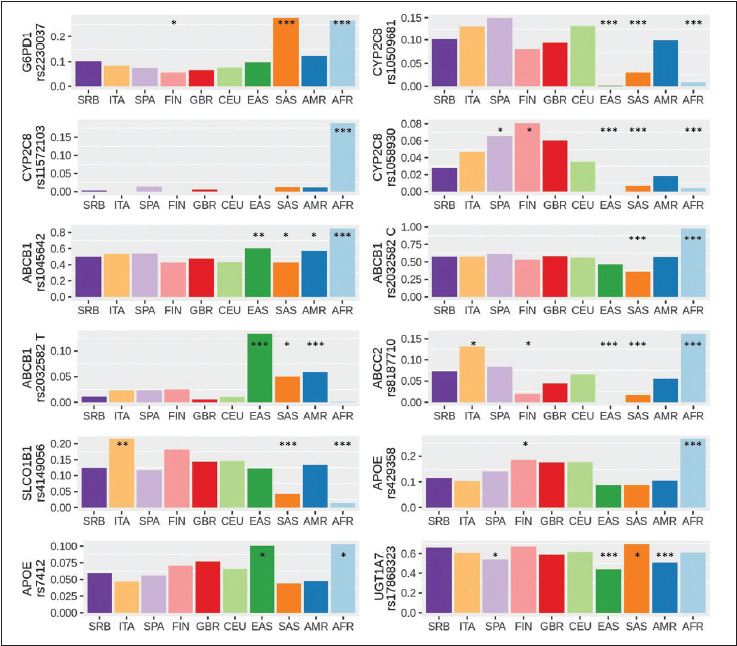
Alternative allele frequency distribution of selected pharmacogenomic variants. Allele frequency of Serbian population was tested against allele frequency of 1000GP populations. One, two and three stars denominate statistically significant difference in allele frequency of other populations in comparison to Serbian populations at the level of p=0.05, 0.01 and 0.001, respectively. SRB–Serbian; ITA–Italian; SPA–Spanish; FIN–Finnish; GBR–British; CEU–Central European; EAS–East Asian; SAS–South Asian; AMR–Central and South American; AFR–African population

Genotype data of healthy individuals from several populations were extracted from 1000GP. Allele frequency of each of 11 variants of interest was presented for Serbian population as well as for other 5 European populations (Italian, Spanish, Finnish, British and Central European from the US) and for populations of East and South Asia, South and Central America and Africa (Table 2). The allele frequency of each 1000GP population was compared to Serbian group and statistically significant differences were noted (Figure 1).

**Table 2:** Allele frequency of Serbian and 1000GP populations. SRB–Serbian; ITA–Italian; SPA–Spanish; FIN–Finnish; GBR–British; CEU–Central European; EAS–East Asian; SAS–South Asian; AMR–Central and South American; AFR–African population

Allele	SRB	ITA	SPA	FIN	GBR	CEU	EAS	SAS	AMR	AFR
G6PD rs2230037 A	10.2%	8.4%	7.5%	5.6%	6.6%	7.6%	9.7%	27.8%	12.4%	26.7%
CYP2C8 rs10509681 C	10.1%	13.1%	15.0%	8.1%	9.3%	13.1%	0.1%	3.0%	9.9%	0.8%
CYP2C8 rs11572103 A	0.3%	0.0%	1.4%	0.0%	0.5%	0.0%	0.0%	1.2%	1.2%	18.9%
CYP2C8 rs1058930 C	2.8%	4.7%	6.5%	8.1%	6.0%	3.5%	0.0%	0.7%	1.9%	0.4%
ABCB1 rs1045642 G	50.0%	53.3%	53.7%	42.4%	47.3%	43.4%	60.2%	42.5%	57.2%	85.0%
ABCB1 rs2032582 C	57.3%	57.5%	61.2%	53.0%	58.2%	56.1%	46.8%	35.8%	57.2%	98.0%
ABCB1 rs2032582 T	1.0%	2.3%	2.3%	2.5%	0.5%	1.0%	13.4%	5.0%	5.9%	0.1%
ABCC2 rs8187710 A	7.3%	13.1%	8.4%	2.0%	4.4%	6.6%	0.0%	1.7%	5.5%	16.2%
SLCO1B1 rs4149056 C	12.4%	21.5%	11.7%	18.2%	14.3%	14.6%	12.3%	4.3%	13.4%	1.4%
APOE rs429358 C	11.5%	10.3%	14.0%	18.7%	17.6%	17.7%	8.6%	8.7%	10.4%	26.8%
APOE rs7412 T	5.9%	4.7%	5.6%	7.1%	7.7%	6.6%	10.0%	4.4%	4.8%	10.3%
UGT1A7 rs17868323 G	62.9%	60.7%	53.7%	67.2%	58.8%	61.6%	43.8%	69.4%	50.7%	61.0%

We have presented our findings in context of drugs used in the treatment of COVID-19 patients.

### Chloroquine/hydroxychloroquine

Four variants in *G6PD* and *CYP2C8* pharmacogenes are possibly associated with chloroquine and hydroxychloroquine response. Allele frequency for the *G6PD* rs2230037 variant, associated with G6PD deficiency and hemolytic anemia, is around 10% for Serbian population, which is higher than in Finnish populations (5.6%), but lower in comparison to South Asians (27.8%) and Africans (26.7%).

Among the three *CYP2C8* variants (rs10509681, rs11572103, rs1058930), associated with decreased *CYP2C8* enzyme activity, the most frequent in Serbian population is *CYP2C8* rs10509681 with AAF of around 10%, followed by rs1058930 variant with AAF of around 3%. These two variants are less prevalent in Asians and Africans. European populations had similar AAF as Serbian regarding rs10509681 and rs1058930, except for Spanish and Finnish that have higher AAF for rs1058930 (6.5% and 8.1%, respectively). Variant *CYP2C* *8* rs11572103 has considerable AAF only in Africans (18.9%).

### ABCB1 variants important for azithromycin, lopinavir and ritonavir pharmacogenomics

Two common variants in *ABCB1* gene, associated with reduced clearance of azithromycin, lopinavir and ritonavir drugs, were included in the analyses. *ABCB*1 rs1045642 G and *ABCB1* rs2032582 C have AAF of around 50% in all European populations, while African population show remarkably high AAF (85% and 98%, respectively). In addition, *ABCB1* rs1045642 G allele is more prevalent in East Asians (60.2%) and South and Central Americans compared to Serbians (57.2%). *ABCB1* rs2032582 T allele is rare in Europeans (0.5-2.5%) and Africans (0.1%), but more common in Asians (5-13.4%) and South and Central Americans (5.9%).

### Lopinavir

Apart from *ABCB1* variants, minor alleles of *ABCC2* rs8187710 and *SLCO1B1* rs4149056 are also associated with reduced clearance of lopinavir. *ABCC2* rs8187710 A allele has frequency of around 7% in Serbian population which is near European average. Italians (13.1%) and Africans (16.2%) have higher AAF, while Asians have lower AAF (0.0-1.7%) compared to Serbian population.

*SLCO1B1* rs4149056 has AAF of around 12% in Serbians. Italians have higher (21.5%), while Africans (1.4%) and South Asians (4.4%) have lower AAF compared to Serbian population.

### Ritonavir

In addition to *ABCB1*, we also analyzed variants in *APOE* and *UGT1A7* pharmacogenes, associated with ritonavir induced dyslipidemia and hyperbilirubinemia, respectively. *APOE* rs429358 C and rs7412 T alleles were found in Serbian population with the frequency of around 12% and 6%, respectively. *APOE* rs429358 C allele is more common in Finnish (18.7%) and African population (26.8%), while *APOE* rs7412 T allele is more common in East Asians (10%) and Africans (10.3%) than Serbians.

*UGT1A7* rs17868323 G allele is common in all analyzed populations with AAF of 62.9% in Serbian population. The Spanish (53.7%), East Asian (43.8%) and South and Central American populations (50.7%) show lower AAF, while South Asians (69.4%) show higher AAF in comparison with Serbian population.

Using an approach of assessment of PGx markers similar to the one used in the study of Serbian population, we have found several variants potentially relevant when specific drugs are administered for treatment of COVID-19 patients worldwide.

As for the European populations, *ABCB1* rs1045642 G and *ABCB1* rs2032582 C variants are rather frequent (around 50 %) in all of them, and could be relevant for reduced clearance of azithro mycin, lopinavir and ritonavir drugs in COVID-19 patients. Additionally, variant *SLCO1B1* rs4149056 is a potential PGx marker of lopinavir in Italian population (level of evidence 2B, predicted protein-damaging effect, AAF 21.5%).

COVID-19 patients in Africa could experience adverse drug response when treated with chloroquine and hydroxychloroquine, since variants *G6PD* rs2230037 and *CYP2C8* rs11572103 have considerable high AAF (26.7% and 18.9%, respectively). Moreover, variants *ABCB1* rs1045642 G and *ABCB1* rs2032582 C are probably PGx actionable markers when azithromycin, lopinavir and ritonavir drugs are used, due to remarkably high AAF (85% and 98%, respectively) in African population. In addition to PGx of lopinavir, *ABCC2* rs8187710 A allele in Africa has the highest AAF worldwide (16.2%).

## Discussion

Population-based genome analysis of specific PGx markers have proven helpful in identifying patients and predicting a higher risk of developing adverse reactions and therefore, leading to a modulation or discontinuation of therapy [37]. Population pharmacogenomics consider significant interethnic differences in the prevalence of PGx markers that have been reported in various studies. A transition from personalized to 'populationalized' medicine and incorporation of pharmacogenomics in public health can benefit in both health-related and economic fields [38].

With no certified therapeutics to efficiently treat COVID-19 patients, repurposing of drugs recognized to be harmless with known pharmacokinetics and optimal dosage could be extremely important. A rational approach to manage COVID-19 pandemic could be to test the effectiveness of the existing antiviral drugs in treating related viral infections. There are more than 160 different therapeutics, already used for treating different diseases and in all phases of development, currently considered for COVID-19 treatment [3] [39], and more than 1450 clinical trials with over 400 of them including drug therapy [40].

Our research effort was oriented towards identifying variant pharmacogenes associated with drugs recommended for COVID-19 treatment, namely chloroquine and hydroxychloroquine, azithromycin, lopinavir and ritonavir, in order to determine Serbian pharmacogenomics landscape and to compare it with other world populations. We have shown that rather frequent PGx markers, *ABCB1* rs1045642 and *ABCB1* rs2032582 (AAF around 50%), could be relevant for reduced clearance of azithromycin, lopinavir and ritonavir drugs and *UGT1A7* rs17868323 (AAF 66.2%) for hyperbilirubinemia in ritonavir treated COVID-19 patients in Serbia. Also, variant *SLCO1B1* rs4149056 is a potential PGx marker of lopinavir response, especially in Italian population with AAF of 21.5%. Our results confirmed that pharmacogenomics landscape of African population is different from the rest of the world.

Chloroquine has already been proven effective *in vitro* against various infections and has been used in treatment of rheumatological and other immunological and neurological diseases [5] [6]. In a clinical study conducted in China involving more than 100 COVID-19 patients, chloroquine was shown to have strong positive effect on both enhanced virus clearance and improved disease outcome [41].

It has been shown that hydroxychloroquine is a more potent inhibitor of COVID-19 virus than chloroquine [15] [42]. With less toxicity and fewer drug-drug interactions than chloroquine, hydroxychloroquine could be the preferred drug over chloroquine [7]. The FDA has issued an emergency use authorization for hydroxychloroquine and chloroquine [43].

Although their toxicity profile is similar, hydroxychloroquine is better tolerated and has a lower incidence of toxicity than chloroquine [16]. The most serious adverse effect includes irreversible toxic retinopathy because it has been shown that chloroquine derivatives bind to melanin, especially the pigmented cells of the eye [44]. The cardiac adverse effect of chloroquine (QTc interval prolongation, Torsade de Pointes, ventricular arrhythmia, and cardiac deaths) has been recognized, especially when patients are treated together with ritonavir and lopinavir, which are also used in COVID-19 therapy [16]. Other adverse effects of chloroquine and hydroxychloroquine include gastrointestinal (nausea, cramps) and cutaneous (rash, itching) manifestations that are considered not to be serious, then, hypoglycemia, and bone marrow suppression with longterm use, but not likely with short-term use [17].

Glucose-6 phosphate dehydrogenase (G6PD) deficient patients are at great risk of developing severe hemolysis after taking chloroquine [18] [19]. G6PD testing before initiation of chloroquine is recommended in COVID-19 patients by NIH Coronavirus Disease 2019 (COVID-19) Treatment Guidelines [45] [46]. There is no evidence that G6PD deficiency is relevant for the use of hydroxychloroquine, and G6PD testing is not recommended.

The efficacy of hydroxychloroquine alone and in combination with the antibiotic azithromycin in reducing the detection of SARS-CoV-2 in patients' upper respiratory tract specimens was demonstrated in two French studies [47] [48]. Another study showed that there is no accelerated viral reduction in COVID-19 patients treated with combination therapy of hydroxychloroquine and azithromycin [49].

Genetic variants in *ABCB1* gene, rs2032582 and rs1045642, could be a cause for decreased concentrations of azithromycin [25]. The same variants are associated with lopinavir/ritonavir response too.

The lopinavir-ritonavir combination of antiviral drugs have shown a clinical improvement in COVID-19 patients, but the literature data are not consistent [14] [20]. However, adverse effect, as significant rise in transaminases, is common in patients receiving this therapy [20] [21], and also dyslipidemia, gastrointestinal upset, hyperbilirubinemia, nephrolithiasis, etc. [22] [23].

Ribavirin is another drug used in treatment of COVID-19 [50]. Although ribavirin pharmacogenomics was not investigated in this study, we find it appropriate to discuss an important PGx variant *IL28B* (*IFNL3*) rs12979860, implicated in therapeutic response of ribavirin used for treating COVID-19. Ribavirin has been used in combination with polyethylene glycol (PEG)ylated interferon-α (PEG-IFN-α/RBV) as standard-of-care therapy for chronic hepatitis C (HCV) treatment [51], but it has been shown that ribavirin could be potentially useful therapeutic for COVID-19 [17] [52].

Adverse effects of ribavirin include hematologic (hemolytic anemia) and liver toxicity. For patients treated with PEG-IFN-α and ribavirin alone, *IL28B* rs12979860 variant, with the level of evidence 1A, is the strongest predictor of treatment response. The rs12979860 CC genotype was associated with a 2.5 or greater rate on average of sustained virological response compared with the TT genotype. TT and CT rs12979860 genotypes have been associated with poor response to ribavirin therapy. It has been shown that allele frequencies of rs12979860 differ worldwide [53]. The frequency of the protective C allele was significantly greater among individuals of European ancestry (80.3%) than those of African ancestry (56.2%) in patients who cleared HCV. Frequency of C allele is in range of 22-44% for African populations, 62-85% for European populations, 70-77% for Southwest Asia, 65-89% for South Asia, 95% for Southeast Asia, 93-100% for East Asia, almost 100% for Oceania, 37-65% for North America and 20-80% for South American populations [53]. Genotype frequency for CC rs12979860 genotype for Serbian HCV patients was 69% [54] which was in concordance with other European populations. However, three more studies on HCV patients in Serbia reported CC rs12979860 genotype frequencies from 25-56% [55] [56] [57]. Considering the fact that allele frequency of therapy non-response associated rs12979860 T allele, vary among populations (from 0% to up to 80%) and could be quite high (40% globally), distribution of rs12979860 genotypes may have an impact on outcome of ribavirin treatment.

To the best of our knowledge, apart from our study, the only study investigating pharmacogenomics landscape of drugs with potential to be used in therapy of COVID-19, taking into account ethnic differences, was reported by Wang and coauthors [58]. Sixty-seven potential drugs for COVID-19 was selected from clinical guideline and clinical trials databases and 313 pharmacogenes related to these therapeutics were included. Genetic variants were analyzed in 125,748 exomes and the expression level of pharmacogenes were evaluated in 17 healthy adults.

The authors have shown that the majority of pharmacogenes mutations (98.52%) were rare (AAF<1%) and non-synonymous (32.8%). Africans had more percentages of common mutations (AAF>1%) comparing to all other populations analyzed. In general, existing CPIC guidelines including ribavirin, α-interferon, chloroquine and captopril could be used, except for African population.

In the same study it was found that two pharmacogenes, *CYP3A4* and *ABCB1*, were affecting the metabolism of about half of drugs investigated. Other pharmacogenes shared with multiple drugs included: *SLC2B1, ALB, CYP3A5, CYP2C9, SLC22A6, SLC22A1, CYP2C19* and *CYP1A2*. Actionable PGx biomarkers identified in the study of Wang and al. are recommended to be tested preemptively. These include: *G6PD* (rs2230037, rs1050828 and rs5030868) for chloroquine hemolysis toxicity, especially for African patients; *VDR* (rs2228570) for ribavirin efficacy in all populations (MAF > 0.5 worldwide); *ITPA* (rs1127354) for ribavirin/α-interferon and anemia risk in Asian patients and ACE (rs1799752) for captopril response in East Asian patients. Moreover, the authors claimed that lopinavir and ritonavir should be carefully utilized in East Asians, since *CYP3A4* (rs28371759) and *ABCB1* (rs2032582) were reported to be with high frequencies. Testing of *ABCB1* gene could be of importance for European (variant rs9282564) and African populations (rs2032582). In addition, treatment with ACE inhibitors could be affected with presence of *ACEII* I/D variants (rs1799752, rs558593002, rs13306087, rs3730025, rs35141294 and rs4314), especially affecting the East Asians. Chloroquine could be the preferred therapy to COVID-19 in European populations with lower frequency of risk alleles [59].

Our study confirmed several findings from the study conducted by Wang et al [58]. Mainly, AAF was found to be very high for variants *G6PD* rs2230037 and *ABCB1* rs2032582 in African population. However, our results point out several novel PGx markers to be considered when COVID-19 therapy is administered.

The presence of significant interethnic differences in the PGx biomarker allele frequencies were confirmed by analyzing individuals from different world populations [60] [61] [62] [63]. Findings that there are remarkable differences in allele frequencies in PGx markers in different geographic areas could be of importance for developing ethnicity-specific guidelines for medical prioritization as well as preemptive testing, especially for the African populations [58] [60]. For ethnically diverse populations the identification of subpopulations with an increased risk of ADRs is also needed [64]. Moreover, when comparing individuals from the same racial group, namely European populations, similar observations were found [62].

The special value of population pharmacogenomics integrated in health care system could be recognized in situations like SARS-CoV-2 pandemic, when there is no specific treatment against the new infectious agent but an urgent need and limited time to treat patients is present. At this moment, no drug has been confirmed to be safe and effective for treating COVID-19. Bearing in mind population specific pharmacogenomics landscape, preemptive testing for pharmacogenes relevant for drugs used in COVID-19 treatment could contribute to better understanding of the inconsistency in therapy response and could be applied to improve the outcome of the COVID-19 patients.

*Acknowledgments*. This work was supported by Ministry of Education, Science and Technological Development Republic of Serbia, EB: 451-03-68/2020-14/ 200042.

## Conflict of interest statement

All the authors declare that they have no conflict of interest in this work.

## List of abbreviations

COVID-19, coronavirus disease 2019;MERS-CoV, Middle East respiratory syndrome coronavirus; SARS-CoV, severe acute respiratory syndrome coronavirus; SARS-CoV-2, severe acute respiratory syndrome coronavirus 2; ADR, adverse drug reactions; ADME, absorption, distribution, metabolism and excretion; PGx, pharmacogenomics; PharmGKB, Pharmacogenomics Knowledgebase; 1000GP, 1000 Genomes Project; TSO, TruSight One; HW, Hardy-Weinberg; AAF, alternative allele frequency; NIH, National Institute of Health; PEG-IFN-α/RBV, polyethylene glycol (PEG)ylated interferon-α/ribavirin; HCV, hepatitis C virus
